# Identifying high risk subgroups of MSM: a latent class analysis using two samples

**DOI:** 10.1186/s12879-019-3700-5

**Published:** 2019-03-05

**Authors:** Smith M. Kumi, Gabriella Stein, Weibin Cheng, William C. Miller, Joseph D. Tucker

**Affiliations:** 1 Division of Epidemiology & Community Health, School of Public Health, University of Minnesota Twin Cities, 1300 South 2nd Street, Minneapolis, MN 55454 USA; 2 Department of Biostatistics, University of North Carolina at Chapel Hill, 135 Dauer Drive, 3101 McGavran-Greenberg Hall, CB #7420, Chapel Hill, NC 27599 USA; 3 Guangzhou Center for Disease Control and Prevention, Department of AIDS/STD Control and Prevention, 1 Jiaochang E Rd, Guangzhou Shi, 510000 Guangdong Sheng China; 4 Division of Epidemiology, The Ohio State University, College of Public Health, 1841 Neal Ave., 302 Cunz Hall, Columbus, OH 43210 USA; 5 Division of Infectious Diseases, University of North Carolina at Chapel Hill, 130 Mason Farm Road, 2nd Floor, Chapel Hill, NC 27599 USA

**Keywords:** LCA, MSM, HIV infection, Vulnerable populations

## Abstract

**Background:**

Latent class analyses (LCA) are increasingly being used to target specialized HIV interventions, but generalizability of emergent population structures across settings has yet to be considered. We compare LCA performed on two online samples of HIV negative Chinese men who have sex with men (MSM) to detect more generalizable latent class structures and to assess the extent to which sampling considerations impact the validity of LCA results.

**Methods:**

LCAs were performed on an 1) nationwide online survey which involved no in-person contact with study staff and a 2) sentinel surveillance survey in which participants underwent HIV and syphilis testing in the city of Guangzhou, both conducted in 2014. Models for each sample were informed by risk factors for HIV acquisition in MSM that were common to both datasets.

**Results:**

An LCA of the Guangzhou sentinel surveillance data indicated the presence of two relatively similar classes, differing only by the greater tendency of one to report group sex. In contrast an LCA of the nationwide survey identified three classes, two of which shared many of the same features as those identified in the Guangzhou survey, including the fact that they were mainly distinguished by group sex behaviors. The final latent class in the nationwide survey was composed of members with notably few risk behaviors.

**Conclusions:**

Comparisons of the latent class structures of each sample lead us to conclude that the nationwide online sample captured a larger, possibly more representative group of Chinese MSM comprised of a larger, higher risk group and a small yet distinct lower group with few reported behaviors. The absence of a lower risk group in the Guangzhou sentinel surveillance dataset suggests that MSM recruited into studies involving free HIV/STI testing may oversample MSM with higher risk behaviors and therefore greater risk perception. Lastly, two types of higher risk MSM were emergent across both samples distinguished largely by their recent group sex behaviors. Higher odds not only of self-reported HIV infection but also of closeted tendencies and gender fluid identities in this highest risk group suggest that interacting factors drive individual and structural facets of HIV risk.

## Background

The established practice in HIV prevention research of subdividing key populations into smaller “risk groups” has been used to prioritize and tailor interventions for groups with specialized needs [1]. Such approaches facilitate effective messaging and program design, especially in populations made up of diverse subgroups such as in men who have sex with men (MSM). Tailoring HIV prevention interventions to specific subgroups of MSM is particularly common and has led to interventions targeting young [2, 3], ethnic minority [4, 5], or drug using [6, 7] MSM. Empirical methods to characterize population heterogeneity are also critical for meaningful modeling of disease dynamics, outcomes of which are highly sensitive to assumptions about population structure and subgroup interactions [8, 9]. These implications demand closer examination of the methods used to identify and characterize these subgroups.

The most common method for subgroup identification involves multiple regression to select variables significantly associated with outcomes of interest, which are then used to delineate the population into levels within the variable, e.g. classifying MSM reporting 10 or more partners in a 6 month period as “high risk” or those with fewer than 10 as “low risk.” [10] Latent class analysis (LCA) has recently emerged as a popular approach to identify subgroups in a given population, favored for its ability to simultaneously consider multiple factors that reveal grouping patterns emergent in the data. LCAs have been used to characterize population structures of various HIV key risk groups such as persons who use illicit drugs [11, 12] or HIV positive individuals [13, 14]. LCAs of MSM are also increasingly common and have examined subgroup structure as it pertains to factors such as sexual HIV risk [15–17], substance use [18], and chronic disease [19].

By shifting focus away from regression models toward methods that account for the co-occurrence of multiple risk factors in individuals, LCAs are believed to achieve greater ecological validity [20, 21] that highlights important interplay among risk factors [22, 23]. The capacity of these methods to inform public health policy, however, requires particular attention to the representativeness of the samples from which inferences are drawn. Analysis of a sample that systematically excludes or over-represents certain segments of the population will distort the completeness of the population structure portrayed. Given the challenges of randomly sampling hard-to-reach populations such as MSM, insights of this population strcture to date are largely informed by proxy methods such as convenience or respondent driven sampling [24] with an increasing number of studies using online methods to recruit and survey participants [25]. In spite of known issues iwth validity and generalizability [24, 26, 27] the convenience of online surveys, coupled with their potential to link subjects to online interventions suggests that we can expect to see more such studies in the future.

Contextualizing the public health insights informed by LCAs must take into account the generalizability of subgroup structures identified when using samples with known biases. To investigate the robustness of group structures identified by LCA models, we conducted the same model analysis on two distinct online samples of Chinese MSM: a survey conducted in a single locale and a nationwide survey. Using LCA with these surveys, we examined HIV-related risk behaviors in uninfected MSM (including those potentially infected but not yet diagnosed) to identify the subgroups based on vulnerability to HIV acquisition. The goal of the comparison across two distinct sampling approaches is to gain insights into the extent to which inferences may be influenced by such details (e.g. study design, recruitment methods, phrasing of questions). Our conclusions also add to existing knowledge regarding the latent structure of Chinese MSM available for online recruitment and provide guidance for future internet-based survey research in these settings.

## Methods

Our analysis was performed on two separate samples of Chinese MSM, one a nationwide survey of MSM recruited online (hereafter the “nationwide online survey”) and the second a city-level HIV sentinel surveillance survey of MSM living in Guangzhou (hereafter the “Guangzhou sentinel surveillance survey”). Details of each survey follow.

### Data sources

The nationwide online MSM survey was conducted in 2014 as part of a trial to assess efficacy of an online intervention to improve HIV testing uptake [28]. In this survey, 1424 men from each of China’s 31 provinces and autonomous regions were recruited and enrolled using banner advertisements on a widely used mobile dating app (BlueD) and a popular online portal for MSM (www.danlan.org). Eligible men were born biologically male, reported ever having had anal intercourse with another man, were at least 16 years of age (the legal age of consent in China), and those willing to provide informed consent. The survey was self-administered through an online platform and thus no biomarker data were collected. The analysis sample was restricted to 1356 participants after removing 68 men (4.7%) with previously diagnosed HIV infection. To further optimize comparability with the Guangzhou sentinel surveillance survey which largely consists of Guangzhou city residents who were all HIV tested as part of their survey participation and lived in an urban area, we excluded another 721(59.7%) MSM from the online survey who indicated that they had never tested, as well as another 53 (4.4%) rural residents. The final analysis sample size included 582 participants.

The Guangzhou survey consisted of data collected during routine HIV sentinel surveillance which is conducted annually by the municipal health department. We restricted our analysis to data collected in 2014 to match the time period of the nationwide online survey. City health authorities oversaw survey implementation which recruits eligible MSM for HIV and STI testing via banner advertisements placed on a popular regional MSM portal largely used for dating, socializing, and sexual health information (www.gztz.org). Men who clicked on the banner were routed through an online appointment making system which provided participants with a choice of three gay-friendly clinics where free testing and counseling are provided. Presenting participants who were eligible and willing to provide informed consent underwent blood testing for HIV and syphilis, results of which were later reported to patients through an online notification system. A questionnaire of demographic and recent sexual behavior information was also collected through self-administered surveys as part of appointment procedures. Out of the 609 men who took part in the survey in 2014, the year selected for this analysis, five (0.68%) were excluded due to a previous HIV diagnosis for a final analysis sample size of 604.

### Statistical analysis

We performed our analyses using PROC LCA [29], a SAS procedure dedicated to latent class analyses, to identify the model with the optimal number of classes based on the most commonly used fit statistics, including the Akaike Information Criterion (AIC) and the sample size adjusted Bayesian Information Criterion (BIC), both for which lower values indicate better fit. Considerations of interpretability and class separation also informed choice of the optimal class number. Latent class model items included the following HIV acquisition risk factors that were available in both of the analysis datasets: 1) more than one sexual partner in the past 6 months [30]; 2) any reporting of recent unprotected anal intercourse (UAI) [31, 32]; 3) preferece as the receptive partner during anal sex (verus inserive; those indicating both positions were classified as receptive preferring) [33]; 4) any reporting of recent group sex [34–37]; 5) age at first sex with another man [38, 39] younger than the median debut age of 20; 6) use of the internet or mobile phone apps as the primary means of seeking sexual partners [40, 41], 7) those indicating "gay" for their sexual orientation (versus straight, bisexual, or “other”), and 8) any reporting of recent drug use (including poppers, ecstasy, methamphetamines, or other recreational drugs) [42–44]. “Recency” of drug use was defined as within the past year for the nationwide online survey, and within the past 6 months for the Guangzhou sentinel surveillance data.

After finalizing the model-identified number of latent classes, we used the PROC LCA *outpost* option to calculate unique and mutually exclusive latent class assignments for every individual in each dataset based on the maximum-probability assignment. We then used binomial and multinomial logistic regression to assess univariable associations between class assignment and odds of key factors unique to each dataset. Key factors available exclusively in the nationwide online survey included the following: identifying as non-male (assessed as whether or not participants responded as “female” or “transgender or transsexual” as opposed to “male” in response to the question "what gender do you currently consider yourself?), gender fluidity (assessed as those who answered “yes” in response to the question, “do you desire a sex change or have you taken steps towards transitioning?”), disclosure of same sex behaviors to medical providers or friends other than same sex partners, and any history of forced sex. Factors available exclusively from the Guangzhou sentinel surveillance dataset included laboratory results HIV and syphilis antibody testing.

### Sensitivity analyses

We performed a sensitivity analyses to examine the effect of our decision to remove over half (59.7%) of the nationwide online survey participants on the basis of their HIV testing history. Sensitivity was assessed both in terms of impact on model fit as well as posterior probabilities of endorsing key items given latent class assignment. A second sensitivity analysis was also performed to examine the composition of a 3-class model in the Guangzhou sentinel surveillance data (our main analysis assumed a 2-class structure for this dataset), given the discordant fit criteria results between a 2 and 3 class model.

## Results

### Study populations

A comparison of the two samples in terms of the response items (Table 1) shows that factors by which the two samples significantly differed included the higher proportions of participants in the nationwide survey who were under age 24 (37.8%; 95% confidence interval [CI], 34.0–41.8% versus 26.8%; 95% CI, 23.4–30.5%), classified as lower income (46.4%; 95% CI, 42.4–50.5% versus 31.8%; 95% CI, 28.2–35.6), who had anal sex with another man before the age of 20 (45.4%; 95% CI, 41.4–49.5 versus 31.4%; 95% CI, 27.5–35.6), and who reported any recent group sex (12.4%; 95% CI, 9.9–15.3 versus 3.8; 95% CI, 2.5–5.9). In the case of education, a higher proportion of the Guangzhou sentinel surveillance sample were classified as being less educated (25.3%; 95% CI, 22.0–29.0 versus 18.6%; 95% CI, 15.6–21.9).

**Table 1:** Prevalence of risk behaviors in the nationwide online survey and Guangzhou sentinel surveillance data, 2014

		Nationwide online survey (*N* = 582)	Guangzhou Sentinel Surveillance (*N* = 604)
		% (95% CI)^a^	% (95% CI)^a^
Demographics			
Age	Under 24	37.8% (34–41.8%)	26.8% (23.4–30.5%)
	25 or older	62.2% (58.2–66.1%)	73.2% (69.5–76.6%)
Education	Less than high school	18.6% (15.6–21.9%)	25.3% (22–29%)
	High school or more	81.4% (78.1–84.4%)	74.7% (71.1–78%)
Marital status	Married	10.5% (8.3–13.2%)	12.1% (9.7–14.9%)
	Not married	89.5% (86.8–91.8%)	87.9% (85.1–90.3%)
Income	<3000RMB a month	46.4% (42.4–50.5%)	31.8% (28.2–35.6%)
	≥3000RMB a month	53.6% (49.6–57.6%)	68.2% (64.4–71.8%)
Behaviors			
Identify as gay^b^	Yes	78.2% (74.6–81.3%)	73.3% (69.7–76.7%)
	No	21.8% (18.7–25.4%)	26.7% (23.3–30.3%)
Main sex partner seeking venue	Online	85.9% (82.9–88.5%)	86.9% (83.9–89.3%)
	Not online	14.1% (11.5–17.2%)	13.1% (10.7–16.1%)
	Missing	0.0	0.3% (0.1–1.2%)
Sexual position	Usually insertive	61.5% (57.5–65.4%)	66.2% (62.3–69.8%)
	Usually receptive	38.5% (34.6–42.5%)	33.8% (30.2–37.7%)
	Missing	0.0	0.7% (0.2–1.7%)
First age of anal sex with another man	< 20 years old	45.4% (41.4–49.5%)	31.4% (27.5–35.6%)
	≥20 years old	54.6% (50.4–58.5%)	68.6% (64.8–72.2%)
	Missing	0.0	16.2% (13.5–19.4%)
Any UAI in the past 6 months	Yes	59.6% (55.6–63.5%)	61.8% (57.8–65.5%)
	No	40.4% (36.5–44.4%)	38.3% (34.5–42.2%)
Any group sex in past 6 months/year^c^	Yes	12.4% (9.9–15.3%)	3.8% (2.5–5.9%)
	No	87.6% (84.7–90.1%)	96.2% (94.3–97.4%)
	Missing	0% (0–0%)	13.7% (11.2–16.7%)
Any drug use in the 6 months/year^c^	Yes	28.4% (24.8–32.1%)	25.3% (22–29%)
	No	71.7% (67.9–75.2%)	74.7% (71.1–78%)

^a^Wald (normal approximation) confidence intervals for proportions were calculated with an alpha level of 5%. For variables with cell counts < 5 Clopper-Pearson confidence intervals were calculated

^b^ Self-reported as “gay,” as opposed to “bisexual,” “straight,” or “other”

^c^ Nationwide survey: within the past year; Guangzhou sentinel surveillance data: within the past 6 months

### Latent class analysis

We compared models with two through six latent classes to identify the optimal fit. Based on AIC and BIC fit criteria (Table 2) as well as considerations of interpretability and class separation, we determined that the three-class model was optimal for the nationwide online survey while the two-class model was optimal for the Guangzhou sentinel surveillance data.

**Table 2:** Fit statistics for latent class models excluding men tested for HIV in nationwide survey

Number of classes	Nationwide online survey (*N* = 582)		Guangzhou City HIV Sentinel Surveillance (*N* = 604)	
	AIC	BIC	AIC	BIC
2	301.10144	375.33144	224.04	298.9008
3	263.07275	376.60098	213.3237	327.8166
4	238.52811	391.35457	218.1502	372.2753
5	248.73276	440.85746	218.8601	412.6174
6	255.6741	487.09703	223.7908	457.1803

Posterior probabilities represent the conditional probabilities of reporting a given behavior given membership in a certain class (Table 3). A probability greater than 50% for a certain item is generally thought to indicate that members of that latent class are more likely to endorse (i.e. to report) that risk factor. Probabilities greater than 50% are marked in bold in Table 3).

**Table 3:** Probabilities of endorsement given latent class assignment, excluding men tested for HIV in nationwide survey

	Nationwide online survey (*N* = 582)			Guangzhou sentinel surveillance survey (*N* = 604)	
	Lowest risk	Moderate risk	Highest risk	Lower risk	Higher risk
	16.1%	66.0%	17.9%	53.6%	46.4%
More than 1 sex partner in past 6 months	**77.5%**	**79.3%**	**74.8%**	**65.5%**	**82.4%**
Any UAI in past 6 months	25.7%	**98.9%**	**92.2%**	**88.2%**	**85.3%**
Usually the receptive partner	**61.9%**	**63.1%**	**55.3%**	**53.8%**	**80.6%**
Any group sex in past year	39.7%	44.0%	**55.9%**	2.6%	**65.4%**
First sex with man before age 20	1.3%	**67.7%**	**82.4%**	**54.8%**	**69.8%**
Finds most partners online	7.8%	2.4%	**53.0%**	3.2%	4.5%
Identifies as gay	8.5%	22.4%	**68.0%**	16.8%	35.2%
Any drug use in the past year	12.8%	**75.8%**	**99.6%**	**54.8%**	**64.5%**

Probabilities greater than 50% indicates that individuals are likely to have reported a given risk factor, and are bolded to facilitate interpretation

This table assumes a 3-class structure for the nationwide survey and a 2-class structure for the Guangzhou sentinel surveillance dataset

In the nationwide online survey, the group that endorsed the greatest number of risk factors made up 17.9% of the sample. This group was named and is hereafter referred to as The Nationwide Highest Risk Class. The class whose members endorsed the fewest risk factors—including having sex with multiple partners in the past 6 months and having a preference for being the receptive partner in anal sex—made up 16.1% of the sample and were therefore designated as “lowest risk” group. The final and largest class (66.0%) was made up of members who endorsed about half of the items and were designated as “moderate risk” class. They departed from the highest risk class in their lower probability of endorsing group sex (44.0% versus 55.9%), online partner seeking (2.4% versus 53.0%), and identifying as gay (22.4% versus 68.0%).

The class breakdown identified by the LCA model for the Guangzhou sentinel surveillance data identified two groups of comparable size (53.6 and 46.4%). Members of each class were likely to endorse nearly all the same items, including multiple sexual partnerships in the past 6 months, any UAI in the past 6 months, preference to be the receptive sexual partner, early debut, and any drug use in the past year. The most notable difference between these two groups was in the tendency for members of the slightly smaller class to report any group sex in the past year (65.4% versus 3.2%), hence our designation of it as the “lower risk” class and the second as the “higher risk” class.

### Associations between latent class membership and key factors

After assigning each study participant to a unique class using the maximum-probability assignment method in PROC LCA, we assessed associations between class assignment and key factors that were only available in one or the other survey.

**Fig. 1:**
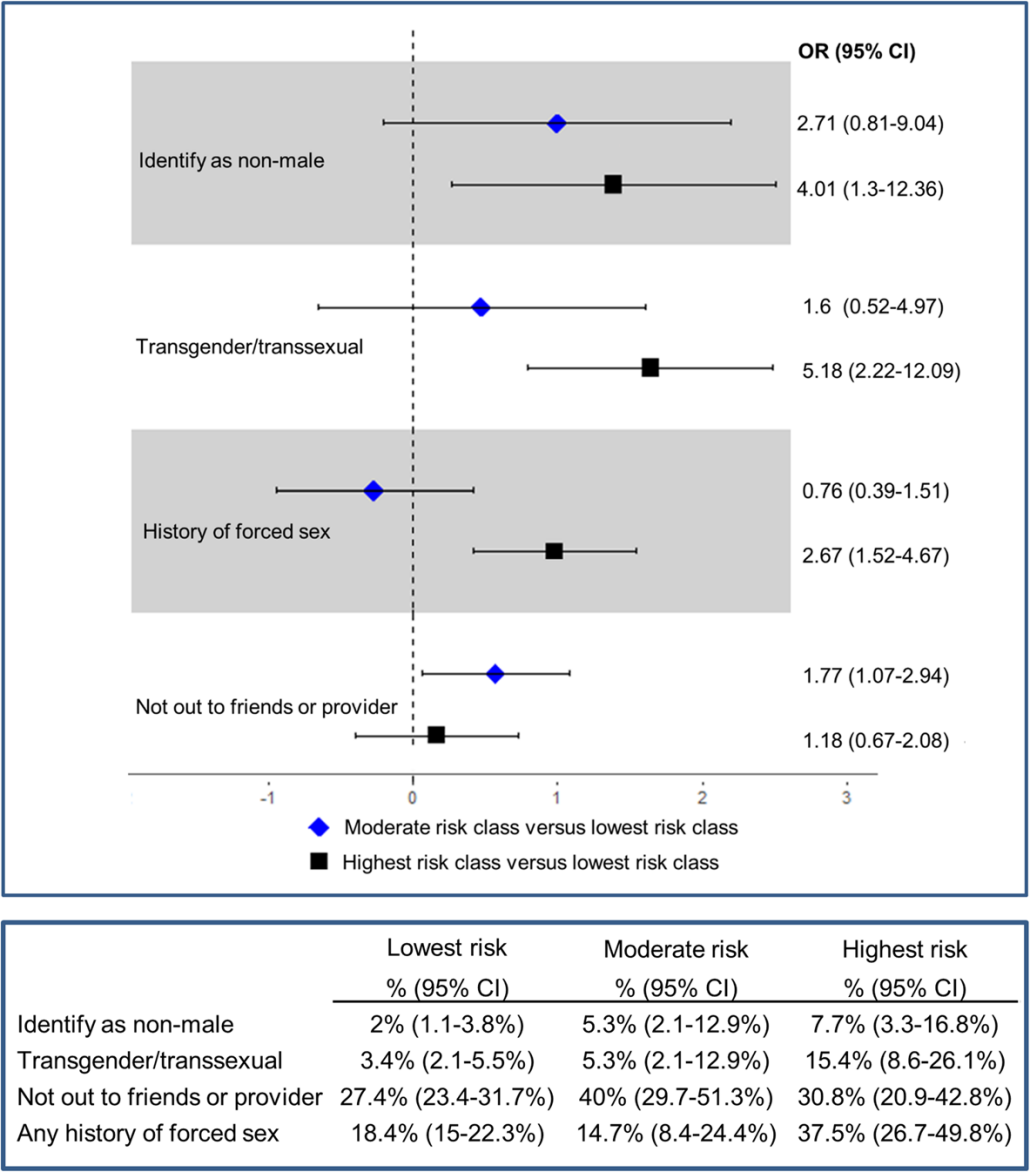
Odds Ratios Comparing Highest and Moderate Risk Classes to Lowest Risk Class. Univariable associations between class membership and key factors in the Nationwide Online Survey (*N* = 703). Designation of the lowest risk class as the referent group is based on the comparatively few reported risk behaviors of members in this class

Of the four psycho-social factors available in the nationwide online survey, the only item that was significantly more common in one class relative to the others was reporting any history of forced sex (37.5%, [95%CI, 26.7–49.8%] in the highest risk class versus 18.4% [95% CI, 15–22.3%] in The Nationwide Moderate Risk Class and 14.7% in the lowest risk class; Fig. 1).

In univariable regression models of the nationwise sample, members of the highest risk class had a greater odds of identifying as non-male (odds ratio [OR]: 4.01, 95% CI,1.30–12.36), of desiring or having taken steps towards transitioning (OR: 5.18; 95% CI: 2.22–12.09) or of having not disclosed their sexual orientation to friends or providers (OR: 2.67; 95% CI: 1.52–4.67), relative to those in the lowest risk class. Members of moderate risk class had greater odds of reporting a history of forced sex (OR: 1.77; 95% CI; 1.07–2.94), relative to those in the lowest risk class (Fig. 1).

**Fig. 2:**
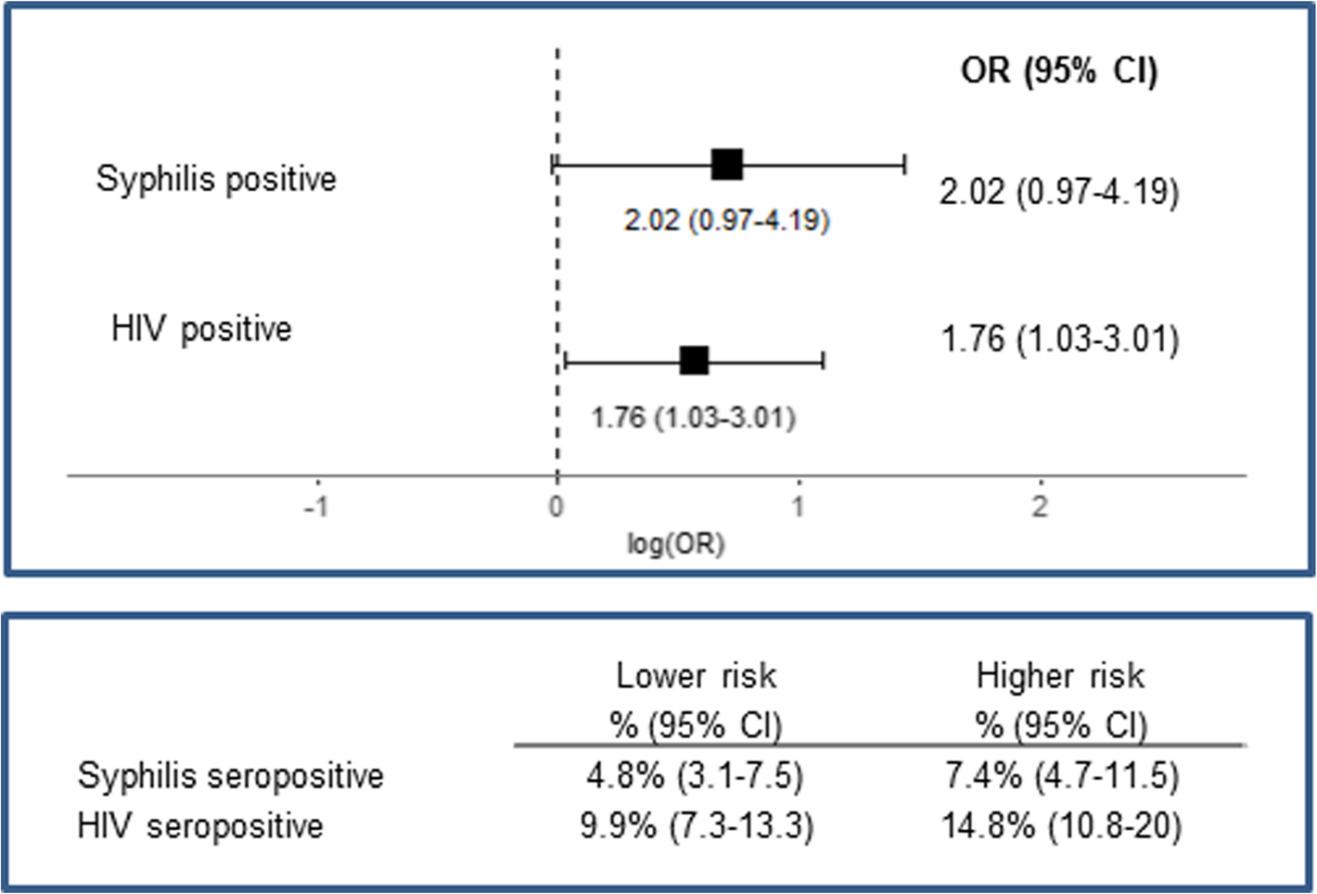
Odds Ratios Comparing High to Lower Risk Class. Univariable associations between latent class membership and key factors in the Guangzhou sentinel surveillance data (*N* = 604). Designation of the lower risk class as the referent group is based on the comparatively few reported risk behaviors of members in this class

In the Guangzhou sentinel surveillance dataset, analysis of biomarkers for HIV and syphilis infection status indicated that prevalence of each was higher in the higher risk class; however, these differences were not statistically significant across classes (Fig. 2).

In univariable regression models of these factors, The Guangzhou Higher Risk Class had significantly higher odds of HIV infection than The Guangzhou Lower Risk Class (OR: 1.76; 95% CI: 1.03–3.01; Fig. 2).

### Sensitivity analyses

In our first sensitivity analysis we examined the impact of our decision to exclude the 721 participants of the nationwide online survey who had never tested for HIV on LCA results (Tables 4 and 5). As the posterior probabilities of the 3-class model that includes the HIV testers indicate, size and composition of each class was largely unaltered, save a few differences (Table 4). Most notably, in the sample that included never-testers, the lowest risk class were more likely to endorse identifying as gay (69.2% versus 8.5%) and less likely to endorse multiple sexual partners in the past 6 months (11.4% versus 77.5%). Similarly those in the moderate risk class were more likely to endorse online partner seeking (99.2% versus 2.4%) and less likely to endorse drug use in the past year (22.7% versus 75.9%).

**Table 4:** Probabilities of endorsement given latent class assignment, including men tested for HIV in nationwide survey (sensitivity analysis)

	Nationwide online survey (*N* = 1207)			Guangzhou sentinel surveillance survey (*N* = 604)	
	Class 1	Class 2	Class 3	Class 1	Class 2
	18.3%	14.1%	67.6%	53.6%	46.4%
More than 1 sex partner in past 6 months	11.4%	**94.7%**	**72.3%**	**65.5%**	**82.4%**
Any UAI in past 6 months	0.3%	**76.1%**	**67.5%**	**88.2%**	**85.3%**
Usually the receptive partner	**62.2%**	50.3%	**65.7%**	**53.8%**	**80.6%**
Any group sex in past year	5.2%	**62.4%**	0.7%	2.6%	**65.4%**
First sex with man before age 20	48.0%	**53.8%**	45.8%	**54.8%**	**69.8%**
Finds most partners online	19.8%	**86.6%**	**99.2%**	3.2%	4.5%
Identifies as gay	**69.2%**	**67.3%**	**75.7%**	16.8%	35.2%
Any drug use in the past year	10.0%	**57.7%**	22.7%	**54.8%**	**64.5%**

Probabilities greater than 50% indicates that individuals are likely to have reported a given risk factor, and are bolded to facilitate interpretation

This table assumes a 3-class structure for the nationwide survey and a 2-class structure for the Guangzhou sentinel surveillance dataset

**Table 5:** Fit statistics for latent class models including men tested for HIV in nationwide survey, were a two-class model to have been assumed (sensitivity analysis)

Number of classes	Nationwide online survey (*N* = 1207)		Guangzhou City HIV Sentinel Surveillance (*N* = 604)	
	AIC	BIC	AIC	BIC
2	422.7336	510.1751	224.04	298.9008
3	323.6194	457.3534	213.3237	327.8166
4	281.9847	462.0113	218.1502	372.2753
5	281.2198	507.539	218.8601	412.6174
6	284.4969	557.1086	223.7908	457.1803

A second sensitivity analysis assessed the composition of a hypothetical 3-class structure in the Guangzhou sentinel surveillance data (our main analysis considered a 2-class model), given the discordant results between the two fit criterion, the BIC and AIC. The resulting 3 classes are made up of one larger and two smaller classes (Table 6), the larger of which is similar in both size (roughly 45%) and endorsement profile as our so-called “higher risk” class identified in the 2-class model. The two remaining classes were made up of a larger class (44.0% of the sample) with a largely similar endorsement profile as the “lower risk” class from the 2-class model, and a smaller class (10.6% of the sample) that differed slightly only in its members’ lower likelihood to endorse multiple sexual partners in the past 6 months (11.6% versus 65.0%).

**Table 6:** Probabilities of endorsement given latent class assignment

	Guangzhou City HIV Sentinel Surveillance (*N* = 604)		
	Class 1	Class 2	Class 3
	10.6%	44.0%	45.5%
More than 1 sex partner in past 6 months	11.6%	**65.0%**	**95.8%**
Any UAI in past 6 months	**64.1%**	**90.5%**	**88.6%**
Usually the receptive partner	**83.1%**	47.3%	**80.6%**
Any group sex in past year	41.0%	3.3%	**57.0%**
First sex with man before age 20	**98.1%**	49.5%	**65.1%**
Finds most partners online	12.2%	3.2%	3.0%
Identifies as gay	28.4%	16.3%	33.4%
Any drug use in the past year	**67.1%**	**53.7%**	**63.3%**

This table assumes a 3-class latent class structure for the Guangzhou sentinel surveillance dataset. Probabilities greater than 50% indicates that individuals are likely to have reported a given risk factor, and are bolded to facilitate interpretation

## Discussion

This study investigated the generalizability of latent class structures identified using LCA by conducting identical analyses on two distinct samples. Differences in the inferred population structures from each sample highlight features of sample design that affect robustness of LCA results. In our LCA analysis of two online samples of Chinese MSM, we identified different numbers of subgroups for each sample. In the nationwide online survey, we identified three classes and in the Guangzhou sentinel surveillance survey we identified two. A closer examination of class composition within each survey suggests that a consistent structure may underlie both samples. This study expands on the existing literature by comparing subgroups based on different samples of MSM recruited using online methods, examining common HIV related risk factors across subgroups.

Among the three classes identified in the nationwide online survey two risk typologies emerge: the lowest risk group with few reported risk behaviors, and the moderate and highest risk classes that both report more UAI and multiple partnerships. Behaviors most useful for distinguishing between the two higher risk groups include group sex and online partner seeking. When examined by factors unique to this survey, we also found that odds of reporting gender fluidity (identifying as a woman or “other”), of having taken steps to transition, and of being closeted to friends or providers were all higher in the highest risk class as compared to the moderate class. The moderate and lower-risk referent classes differed only terms of the fact that the former was significantly more likely to report a history of forced sex.

In our examination of the Guangzhou sentinel surveillance survey, only one typology emerged from the two very similar “higher risk” classes. Similar to the two riskier classes observed in the nationwide survey, each of these two classes differed most notably in terms of reported group sex behaviors. Associations between latent class assignment and biological outcomes also suggest that risk of HIV infection is likely higher in the higher risk class.

Comparisons in the latent class structures of the two samples therefore lead us to the following conclusions: 1) the presence of a sizable and distinctly lower risk class in the nationwide online sample likely explains the difference in the observed latent class structures between the LCAs conducted on the nationwide online survey and the Guangzhou sentinel surveillance survey, and 2) a common features of both LCA results was the presence of a small, highest risk group in each sample defined largely by their tendency to endorse group sex.

Presence of a lower risk class in the nationwide online survey suggests differential sampling bias across the two surveys, likely due to different motivations for taking part in each survey. That is, participants in the Guangzhou sentinel surveillance participants must undergo clinic based HIV/STI testing as part of their participation in the survey. In contrast, participants of the nationwide online survey simply filled out surveys on their own electronic devices without any direct contact with study staff. As such, the presence of a large lower risk group in the nationwide survey that is absent from the Guangzhou sentinel surveillance data may indicate the role that differences in recruitment methods and participants’ willingness to test shape the composition of each sample. Though motivations for undergoing HIV/STI testing were not asked of the Guangzhou sentinel surveillance participants, reasons cited by Chinese MSM in other similar studies suggests that factors such as recent sexual exposures [45] or a perceived need for testing [46] may play a role. Lower risk individuals aware of the Guangzhou survey may have abstained from participating if they perceived themselves to be of lower risk or if they had fewer recent sexual exposures about which they were concerned.

Findings presented here must be interpreted in light of several limitations. These data provide useful insights into the population of Chinese MSM available for recruitment online; however, generalizability of findings from either sample can in no way extend to the entirety of the Chinese MSM population. More research is needed to understand the extent and patterns of representativeness of MSM willing and interested in being recruited online. Field outreach by Guangzhou disease control authorities has recently identified subgroups of MSM who have never participated in their sentinel surveillance studies, who largely find partners at cruising sites in public parks, restrooms, or gay social clubs (paid entry venues where MSM socialize and meet new sexual partners). A recent pilot study of men at one social club reported an alarmingly high HIV prevalence rate of 25.9% [47]. The fact that most of the men were of lower socioeconomic status and that few had previously tested for HIV suggests that the current online approach to conducting sentinel surveillance may be systematically overlooking this high risk group.

Another limitation entails our inability, due to the study design, to verify that participants in the Guangzhou sentinel surveillance study are truly made up of those currently living in Guangzhou. However the regionally based recruitment campaign is believed to have mitigated large numbers of enrollees from outside the region from taking part in the survey.

Findings from this study suggest that most MSM populations recruited via online methods in this setting have sufficiently high risk behaviors to merit interventions tailored to their particular needs. Within populations recruited online, however, those reporting a history of group sex may a key subset to target for specialized interventions addressing the elevated HIV acquisition risk associated with this behavior. In addition, the higher odds of HIV infection in the highest risk class in the Guangzhou sentinel surveillance data, as well as the higher odds among higher risk class members for being closeted or having a gender fluid identify all suggest that sexual HIV risk concentrated in this subset co-occurs with other factors that further contribute to their marginalization. Interventions to address the health needs of vulnerable MSM may therefore benefit from a holistic approach to addressing the multifaceted and potentially interacting sources of risk faced by these individuals [22, 48].

Future LCA to determine the latent construct of key populations may benefit from comparing latent class structures identified in more than one sample of the study population. Such an approach may identify previously undetected latent classes if samples captured a previously excluded subgroup. Discrepant class identification across different samples can also provide critical insight into the generalizability of findings from a single LCA, and highlight key recruitment and design features of studies that may affect sample composition. For example, the Guangzhou sentinel surveillance survey may benefit in future rounds of recruitment by adding screening questions for non-participants in order to better understand differences between eligible non-participants and those who ultimately enroll and undergo HIV testing.

## Conclusions

Combining results from two simultaneous LCAs conducted on distinct samples of Chinese MSM provided more robust insights than would have been possible from a single LCA. Results presented here may serve as a template for future LCAs but also catalyze greater reflection among public health researchers regarding ways to strengthen our methodological approaches to mapping and characterizing HIV risk.

## Abbreviations

AIC: Akaike Information Criterion
BIC: Bayesian Information Criterion
LCA: Latent Class Analysis
MSM: Men who have Sex with Men
UAI: Unprotected Anal Intercourse
