# Correction to: Identifying high risk subgroups of MSM: a latent class analysis using two samples

**DOI:** 10.1186/s12879-019-3886-6

**Published:** 2019-03-26

**Authors:** M. Kumi Smith, Gabriella Stein, Weibin Cheng, William C. Miller, Joseph D. Tucker

**Affiliations:** 1 Division of Epidemiology & Community Health, School of Public Health, University of Minnesota Twin Cities, 1300 South 2nd Street, Minneapolis, MN 55454 USA; 2 Department of Biostatistics, University of North Carolina at Chapel Hill, 135 Dauer Drive, 3101 McGavran-Greenberg Hall, CB #7420, Chapel Hill, NC 27599 USA; 3 Department of AIDS/STD Control and Prevention, Guangzhou Center for Disease Control and Prevention, 1 Jiaochang E Rd., Guangzhou Shi, 510000 Guangdong Sheng China; 4 Division of Epidemiology, The Ohio State University, College of Public Health, 1841 Neal, Ave., 302 Cunz Hall, Columbus, OH 43210 USA; 5 Division of Infectious Diseases, University of North Carolina at Chapel Hill, 130 Mason Farm Road, 2nd Floor, Chapel Hill, NC 27599 USA

**Correction to: BMC Infect Dis (2019) 19:213**

**https://doi.org/10.1186/s12879-019-3700-5**

Following publication of the original article [1], the author reported his family name has been marked as the first name. His given name is M. Kumi and his family name is Smith.

The original article has been corrected as well.

The publisher apologizes for any inconvenience caused by this error.
